# Reverse sheathing technique for iliocaval thrombectomy in the setting of IVC filters

**DOI:** 10.1186/s42155-024-00437-7

**Published:** 2024-02-22

**Authors:** Joshua Cornman-Homonoff, Angelo G. Marino, Hamid Mojibian

**Affiliations:** Department of Radiology and Biomedical Imaging, Section of Interventional Radiology, Yale University School of Medicine, 330 Cedar Street, TE 2-205, New Haven, CT 06510 USA

**Keywords:** Iliocaval thrombosis, DVT thrombectomy, IVC filter

## Abstract

The Inari ClotTriever system (Inari Medical, Irvine, California) is safe and effective for the treatment of DVT. However, because it consists of a 31 cm coring device and collection bag that must be extended for use, application may be precluded by available intravascular “running room”, such as in the presence of an IVC filter. Here we present a technique for bypassing IVC filters via retrograde deployment of the ClotTriever within a sheath, as illustrated in three cases. This technique extends the applicability of the ClotTriever to locations in which its length would otherwise preclude use.

## Background

The Inari ClotTriever system (Inari Medical, Irvine, California) is safe and effective for the treatment of DVT [1, 2]. However, because it consists of a 31 cm coring element and collection bag that must be extended for use, application may be precluded by available intravascular “running room”. One scenario in which this limitation presents itself is in the presence of an IVC filter, which could catch on the device. Here we present a technique for bypassing IVC filters via retrograde deployment of the ClotTriever within a sheath, as illustrated in three cases. IRB exemption was granted for this series.

## Results

### Case 1

**Fig. 1:**
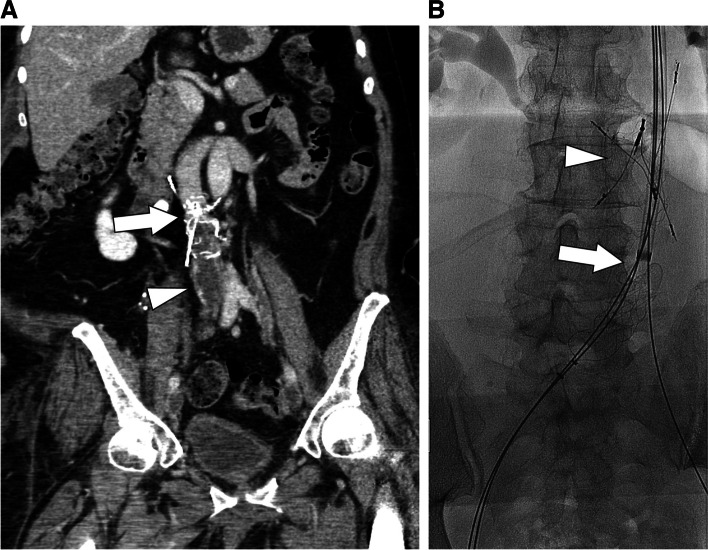
**a**. Coronal CT image demonstrating the Bird’s Nest filter (arrow) with thrombus extending peripherally below it (arrowhead). **b**. Frontal fluoroscopic image showing the ClotTriever deployed inside the right internal jugular sheath (arrow), avoiding the filter (arrowhead)

An 84-year-old woman who underwent meningioma resection 20 years prior preceded by prophylactic placement of a Bird’s Nest filter (Cook Medical, Bloomington, Indiana) presented with bilateral lower extremity edema and was found to have occlusive thrombus extending from the filter into the bilateral popliteal veins (Fig. 1a). Systemic thrombolysis was felt to be high risk given her age, so mechanical thrombectomy was performed. With the patient supine, a 14 French sheath was introduced in the right internal jugular vein (IJV) and a suprarenal filter placed as embolic protection. An AngioJet catheter (Boston Scientific, Marlborough, Massachusetts) was then used to infuse 10 mg of tissue plasminogen activator into the IVC and both lower extremities. The patient was then repositioned prone and 16 French ClotTriever sheaths placed in both popliteal veins. After achieving through-and-through access, the IJ sheath was advanced below the level of the filter. The ClotTriever was then sequentially introduced through each popliteal vein and advanced into the IJ sheath, deployed with the coring element just outside of the sheath and the collection bag within it, and then retracted toward the popliteal access site (Fig. 1b). After reestablishing patency and dilating the central deep veins, iliocaval stenting was performed.

### Case 2

**Fig. 2:**
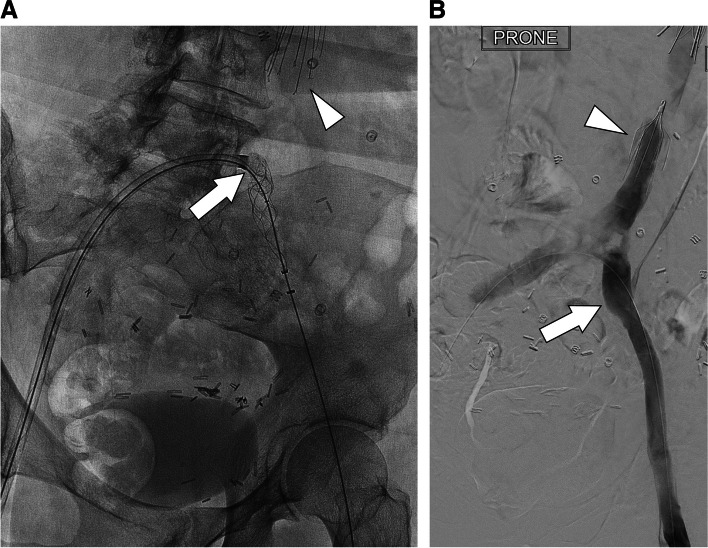
**a**. Frontal fluoroscopic image showing the ClotTriever deployed inside the popliteal sheath across the iliocaval bifurcation (arrow), avoiding the filter (arrowhead). **b**. Frontal digital subtraction angiogram demonstrating improved patency of the lower extremity deep veins (arrow) and IVC below the level of the filter (arrowhead)

A 75-year-old woman with breast cancer, malignant gastrointestinal stromal tumor, and prior venous thromboembolism on Lovenox presented with a headache and was found to have hemorrhagic right dural metastases. Anticoagulation was discontinued and an infrarenal IVC filter placed. Three months later she presented with right lower extremity edema and was found to have DVT extending from the filter through the popliteal vein on the right and the superficial femoral vein on the left. Thrombolytics were contraindicated given her age and intracranial metastases, so mechanical thrombectomy was performed. With the patient supine, a suprarenal filter was placed as embolic protection. Then, with the patient prone, a 12 French sheath was placed in the left popliteal vein and a 13 French ClotTriever sheath in the right. A Glidewire Advantage guidewire (Terumo, Shibuya, Tokyo, Japan) was snared from the right popliteal sheath out the left, establishing through-and-through access. The ClotTriever device was then introduced through the right popliteal sheath into the left popliteal sheath and deployed (Fig. 2a). Multiple passes were then made, reestablishing patency of the lower extremity deep veins (Fig. 2b). Thrombus was removed from the filter using a FlowTriever catheter (Inari), after which angioplasty and iliocaval stenting were performed.

### Case 3

**Fig. 3:**
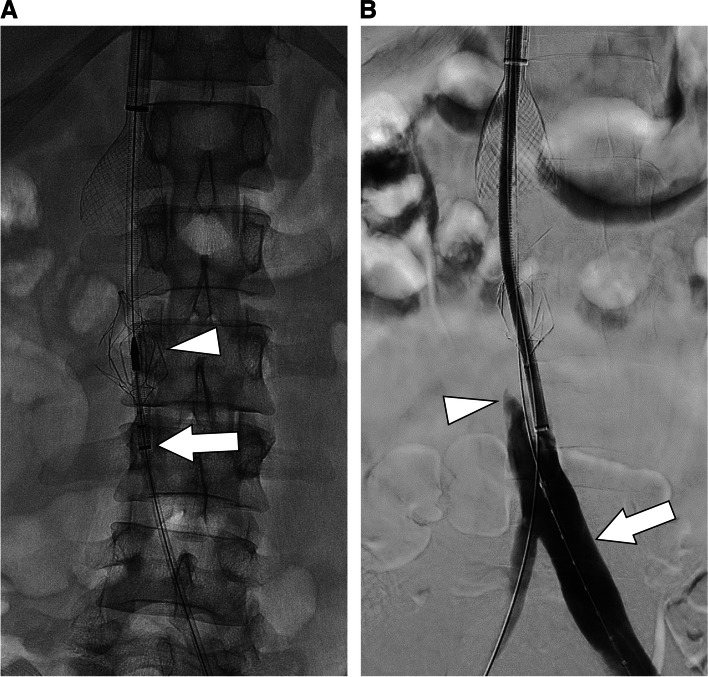
**a**. Frontal fluoroscopic image showing the Protrieve sheath with coaxial 12 French sheath through it (arrow). The tip of the undeployed ClotTriever is visible within the sheath (arrowhead) at the level of the filter. **b**. Frontal digital subtraction angiogram demonstrating improved patency of the lower extremity deep veins (arrow) with persistent thrombus below the filter (arrowhead)

A 34-year-old male with Factor V Leiden, recurrent DVT despite anticoagulation, and an indwelling fractured TRAPEASE IVC filter (Cordism Hialeah, Florida) who had undergone multiple prior thrombectomies presented with shortness of breath and lower extremity swelling. He was found to have subsegmental pulmonary emboli and thrombus extending from the filter through both lower extremities, so mechanical thrombectomy was performed. With the patient supine, a 20 French Protrieve sheath (Inari) was introduced into the right IJV to act as embolic protection. A 16 French ClotTriever sheath (Inari) was placed in the left popliteal vein and through-and-through wire access obtained. A 12 French sheath was then introduced coaxially through the Protrieve sheath and advanced until the tip was caudal to the filter. The ClotTriever was then advanced via the popliteal access into it the 12 French sheath, deployed, and mechanical thrombectomy performed (Fig. 3a, b). Additional thrombus was removed from the IVC and right leg with a FlowTriever aspiration catheter (Inari), after which a stent was placed across the fractured filter. A retrievable IVC filter was then placed in a suprarenal position.

## Conclusions

ClotTriever use is limited by the length of the collection basket, which requires adequate room for extension. As shown here, additional length can be generated through introduction of a sheath at the far end of the device, into which the basket can be advanced. The sheath must be a minimum of 12 French and is most effective when through-and-through wire access is present. Although the Protrieve sheath is large enough to accommodate the basket, the sheath funnel can become entangled with it and so should only be used with a coaxial sheath present. In conclusion, the reverse sheathing technique extends the applicability of the ClotTriever to locations in which its length would otherwise preclude use.

## Data Availability

Not applicable.
